# Identification of nine cryptic species of *Candida albicans*, *C. glabrata*, and *C. parapsilosis* complexes using one-step multiplex PCR

**DOI:** 10.1186/s12879-018-3381-5

**Published:** 2018-09-25

**Authors:** Amir Arastehfar, Wenjie Fang, Weihua Pan, Wanqing Liao, Liang Yan, Teun Boekhout

**Affiliations:** 1 Westerdijk Fungal Biodiversity Institute, Utrecht, 3584 the Netherlands; 2 Department of Dermatology, Shanghai Key Laboratory of Molecular Medical Mycology, Shanghai Institute of Medical Mycology, Shanghai Changzheng Hospital, Second Military Medical University, Shanghai, 200003 China; 3 Institute of Biodiversity and Ecosystem Dynamics, University of Amsterdam, Amsterdam, 1012 WX the Netherlands

**Keywords:** Cryptic species, *Candida*, Molecular diagnosis, Multiplex PCR

## Abstract

**Background:**

*Candida albicans*, *Candida glabrata*, and *Candida parapsilosis* are three prevalent causes of candidiasis, worldwide. These species are considered as nine medically important complex species. Limited knowledge about these newly recognized species prompted us to develop a one-step, multiplex PCR to detect and identify them in clinical settings.

**Methods:**

Primers targeting Hyphal Wall Protein I gene for the *C. albicans, C. dubliniensis, C. africana*, Intergenic Spacer for the *C. glabrata, C. nivariensis, C. bracarensis*, and Intein and ITS rDNA for the *C. parapsilosis, C. orthopsilosis,* and *C. metapsilosis* were designed. Using 168 CBS reference strains and 280 clinical isolates, the specificity and reproducibility of the developed assay were evaluated.

**Results:**

Our developed assay successfully identified and distinguished all the nine species. No cross-reaction with closely- and distantly-related yeast species, *Aspergillus* species and human DNA was observed, resulting in 100% specificity. The ambiguous results obtained by MALDI-TOF for *C. albicans* and *C. africana* were corrected by our 9-plex PCR assay. This assay identified all the cryptic complex species from two test sets from Iran and China, correctly.

**Conclusions:**

Our developed multiplex assay is accurate, specific, cost/time-saving, and works without the tedious DNA extraction steps. It could be integrated into routine clinical laboratories and as a reliable identification tool and has the potential to be implemented into epidemiological studies to broaden the limited knowledge of cryptic species complexes.

**Electronic supplementary material:**

The online version of this article (10.1186/s12879-018-3381-5) contains supplementary material, which is available to authorized users.

## Introduction

*Candida albicans*, *C. glabrata*, and *C. parapsilosis* were reported to be the three most clinically important *Candida* species [1]. Due to the inclusion of newly designated species through taxonomic studies, these three *Candida* species are now considered cryptic complex species, including *C. albicans*, *C. africana*, and *C. dubliniensis* (as *C. albicans* complex) [2, 3], *C. glabrata*, *C. nivariensis*, and *C. bracarensis* (as *C. glabrata* complex) [4, 5], and *C. parapsilosis*, *C. orthopsilosis*, and *C. metapsilosis* (as *C. parapsilosis* complex) [6]. Despite the fact that for more than a decade these cryptic species have been introduced, still limited knowledge exists on their distribution, pathogenicity, and antifungal susceptibility pattern. Moreover, from an evolutionary standpoint, identification and discrimination of these cryptic species complexes could shed light on pathogenicity acquisition, as Pryszcz et al. (2015) found *C. metapsilosis* as a highly heterozygous opportunistic pathogen arose from a two parental lineages that were not pathogenic [7].

Biochemical and morphological tests failed to unequivocally identify and differentiate these cryptic species, which is attributed to the presence of similar phenotypic properties among the complexes [8]. Accordingly, various methodologies, including amplified fragment length polymorphism (AFLP) [9], matrix-assisted laser desorption-time of flight (MALDI-TOF) [10], sequencing of ITS rDNA [11] have been used to tackle this problem. Nowadays, due to affordability and the high reproducibility of PCR, myriad aspects of biology have been revolutionized throughout the world [12, 13]. In line with this, identification and differentiation of *Candida albicans*, *Candida glabrata*, and *Candida parapsilosis* complexes representatives through fast and simple conventional PCR by targeting hyphal wall protein 1(HWP1) gene [14], large ribosomal protein 31 (RPL31) gene [15], and vacuolar ATPase (VMA) gene [16], have been addressed. However, the simultaneous detection of all of aforementioned nine medically important cryptic species in a single reaction was not described, previously.

Consequently, the aim of this study was to develop an easy-to-perform, low-cost, highly specific and accurate, multiplex PCR assay capable of differentiation of all these nine medically important *Candida* species, without tedious DNA extraction steps and directly through mixing of pure colonies into the PCR master mix.

## Materials and methods

### Isolates and growth conditions

Four hundred forty-eight isolates including, 168 reference strains (111 target cryptic strains and 57 non-target strains representing 57 species) from Westerdijk Fungal Biodiversity Institute (Additional file 1: Table S1) and 280 clinical isolates comprising only cryptic species complexes (Table 1) were included. Chinese clinical isolates (*n* = 145) were pooled from a Chinese collection (*n* = 1500), and included cryptic species of *C. glabrata* and *C. parapsilosis* complexes and the Iranian clinical isolates (*n* = 135) included cryptic species complexes of *C. albicans*. Clinical isolates were from different anatomical sites, ranging from nail, sputum, urine and bronchial fluids to blood and were collected from different hospitals in Iran and China (Table 1). CBS reference strains were used for optimization and specificity testing, while clinical isolates used for confirming reproducibility of the developed multiplex PCR assay. Isolates were grown on Glucose Yeast Extract Peptone Agar (Westerdijk Fungal Biodiversity Institute, Utrecht, Netherlands) at 25 °C for 48 h.

**Table 1:** Clinical isolates utilized for reproducibility and validation testing

Species	Number of Isolates	Origin
Iran (*n =* 135)		
*C. albicans*	128	Urine, blood
*C. africana*	3	Vagina
*C. dubliniensis*	4	Urine
China (*n =* 145)		
*C. nivarensis*	2	Sputum
*C. glabrata*	87	Urine, blood, sputum, BALF^a^
*C. metapsilosis*	20	Sputum
*C. orthopsilosis*	6	Nail, blood, mouths
*C. bracarensis*	2	Sputum
*C. parapsilosis*	28	Urine, blood, nail, sputum

^a^*BALF* bronchoalveolar lavage fluid

### DNA extraction

DNA extraction was carried out using CTAB and phenol-chloroform method as described previously [17]. Briefly, a full loop of fresh yeasts colonies was suspended in 700 μL of CTAB buffer followed by bead beating (TissueLyzer II, QIAGEN, Hannover, Germany) for 3 min, 3000 beats/minute. After incubation for 60 min at 55 °C, 700 μL of phenol-chloroform was added. Upon vortexing and centrifugation for 20 min at 14000 rpm, 4 °C, 400 μL of supernatant was added to isopropanol. Finally, upon washing with 70% ethanol and drying the DNA samples on air, the pellets were suspended in Tris-EDTA (10 mM Tris Base, 1 mM EDTA, pH 8.0) buffer. DNA purity and quantity was assessed using NanoDrop and Qubit Broad range kit (Invitrogen) and the quality was evaluated by electrophoretic separation of 5 μl of DNA samples on 1% agarose gel.

### Primer design

**Fig. 1:**
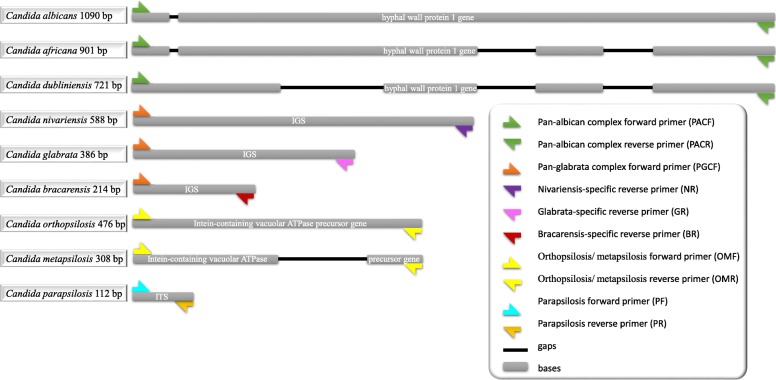
Primer information. List of covered species, their PCR product sizes, and their locations are depicted

Target sequences were retrieved from NCBI website (https://blast.ncbi.nlm.nih.gov/Blast.cgi). Primers utilized in this study were all designed by authors, and are listed and depicted in Table 2 and Fig. 1. Primers were selected if 1) lack of cross-reactivity with each other and other non-target species, 2) Compatible PCR product size, 3) compatible in Tm values, and 4) positioned in the most stable part of target loci. Gaps and mismatches with non-target species were located in the 3^΄^ end of primers, hence allow specific amplification of target species. Online free software of Integrated DNA Technology was used to calculate Tm and Delta G of primers (https://eu.idtdna.com/calc/analyzer).Primers were manufactured by Integrated DNA Technology (IDT) Company.

**Table 2:** Sequence of utilized primers in this study

Primer Name	Primer sequences	Annotation
PACF	GCTACCACTTCAGAATCATCATC	Universal forward (PACF) and reverse (PACR) primers for *C. albicans, C. dubliniensis and C. africana*
PACR	AGATCAAGAATGCAGCAATACCAA	
PGCF	TCACTTTCAACTGCTTTCGC	Universal forward primers for *C. glabrata, C. nivariensis* and *C. bracarensis*
GR	TGCGAGTCATGGGCGGAA	Reverse primer only for *C. glabrata*
NR	ACCCCAGAGGCATAAATAGC	Reverse primer only for *C. nivariensis*
BR	GCAACTGGACGAAAGTGC	Reverse primer only for *C. bracarensis*
PF	GCGGAAGGATCATTACAGAATG	Forward (PF) and reverse (PR) primers specifically for *C. parapsilosis*
PR	CTGGCAGGCCCCATATAG	
OMF	GAGAAAGCACGCCTCTTTGC	Universal forward (OMF) and reverse (OMR) primers for *C. orthopsilosis* and *C. metapsilosis*
OMR	TCAGCATTTTGGGCTCTTGC

### PCR condition

The PCR reaction was optimized in a final volume of 50 μL as follows: 37 μL MiliQ water (Merck Millipore, Billerica, Massachusetts, United States), 5 μL 10X buffer, 1.5 mM MgCl_2_, 2.5 units of *Taq* enzyme (Bio Taq DNA Polymerase, Biolab), 0.2 mM of mixed dNTP (dNTP mix, 100 Mm, Biolab), *C. albicans* complex primers pair 10 p-mole each one, 5 p-mole for the rest of the primers (*C. glabrata* and *C. parapsilosis* complex primers), and 1 μL of DNA template.

PCR (2720 Thermal Cycler,, Applied Biosystems, Waltham, Massachusetts, USA) used the following program, pre-denaturation for 5 min at 94 °C, 35 cycles of 94 °C for 30 s, 60 °C for 30 s, 72 °C for 30 s, and final extension at 72 °C for 8 min. PCR products were run on 2% agarose gel for 70 min (8 Volt/cm), stained with GelRed (BioTium Corporation, USA) and visualized using gel documentation with exposure time of 4 s (Gel Doc XR^+^, BioRad, California, USA). In order to prepare in-house ladders, 10 μL of PCR products from controls (90 μL), 10 μL deionized water, and 20 μL of 10X loading dye were mixed. 15 μL of prepared in-house ladders were utilized as marker to aid in identifying target species (Additional file 2: Figure S1).

### Optimization of 9-plex PCR assay using CBS reference strains

After initial successful amplification from CBS 2691 (*C. albicans*), CBS 7987 (*C. dubliniensis*), CBS 8781 (*C. africana*), CBS 138 (*C. glabrata*), CBS 9983 (*C. nivariensis*), CBS 10154 (*C. bracarensis*), CBS 11045 (*C. parapsilosis*), CBS 10906 (*C. orthopsilosis*) and CBS 2916 (*C. metapsilosis*), A panel of 111 CBS reference strains (Additional file 1: Table S1) encompassing only cryptic species of *C. albicans*, *C. glabrata*, *C. parapsilosis* complexes were further tested with our 9-plex PCR.

### Specificity testing

To evaluate the specificity of the 9-plex PCR assay, 100 DNA samples containing 57 closely- and distantly-related yeast species, *Aspergillus* spp., and human DNA (from blood) and 43 target strains pooled from CBS reference (optimization test set) were prepared in a blinded fashion (blind test set) (Table 1). For preparation of the specificity test set, two technicians were involved. The first technician randomly dispersed DNA samples and serially coded them from one to 100. The second technician subjected the specificity blind test set to the optimized multiplex PCR. The first technician was provided with the results derived from PCR and the consistency of the results was checked with the CBS identity of each strain.

### Pure colony testing

In order to eliminate the DNA extraction step and confirm the compatibility of the multiplex assay, single colonies of yeast species derived from the blind test set were subjected to PCR as a template (except for *Rhodotorula mucilaginosa*). Briefly, a single colony (≈ 1 mm^3^) was suspended in the prepared PCR master mix (50 μl) as mentioned earlier.

### Validation of 9-plex multiplex PCR using clinical isolates

Upon optimization of the multiplex PCR assay using CBS reference strains, specificity and pure colony testing, a panel of clinical isolates (*n =* 280) (Table 1) prepared in a blind fashion were subjected to the 9-plex assay. One technician performed the LSU sequencing and MALDI-TOF. Upon, identification, the first technician coded the yeast culture plates from 1 to 280, and then a new random number (integer number from 1 to 280) for each plate was generated by SPSS random number generator (version 21, International Business Machines Corp, Armonk, New York, United States) and used as blind test number. The second technician subjected the blind test set to the optimized multiplex PCR. The first technician was provided with the results derived from PCR and the consistency of these results with MALDI-TOF and sequencing were evaluated.

### MALDI-TOF

The full extraction method using MALDI-TOF MS (Bruker Biotyper, MicroFlex, LT, Bruker Daltonics, Bremen, Germany) was followed, according to the manufacturer’s instructions (https://www.bruker.com/fileadmin/user_upload/8-PDF-Docs/Separations_MassSpectrometry/InstructionForUse/IFU_268711_267615_226413_MALDI_Biotarget_48_Rev1.pdf). Briefly, after suspending of pure colonies in 300 μL of MiliQ water, 900 μL of absolute ethanol was added. After centrifugation step (14,000 rpm, 3 min, room temperature) the supernatant was discarded and another centrifugation step was repeated to totally discard the supernatant. Subsequently, 70% formic acid (Sigma Aldrich, St. Louis, Missouri, United States) was added and upon five minutes incubation at room temperature equal amount of Acetonitrile was added. After a final centrifugation step (14,000 rpm, 3 min, room temperature) 1 μL of clear supernatant was transferred onto the 96-well target plate. Subsequently, 1 μL of matrix was overlaid on the top of air dried samples. Finally, the 96 well target plate was loaded into the MALDI-TOF device.

### Sequencing

In order to ensure that the designed primers targeted the right genes, the amplified PCR products were subject to bidirectional dideoxy chain terminated Sanger sequencing using the respective primers. Obtained sequences were subjected to online searching database of NCBI (https://blast.ncbi.nlm.nih.gov/Blast.cgi).

The ribosomal DNA large subunit (LSU) primers including LROR (5΄-ACCCGCTGAACTTAAGC-3΄) and LR5 (5΄-TCCTGAGGGAAACTTCG-3΄) were exploited to address the identity of each isolate [18]. Sequences obtained for each isolate was subject to BLAST online database tool (https://blast.ncbi.nlm.nih.gov/Blast.cgi).

## Results

### Development of 9-plex PCR assay using CBS reference strains

**Fig. 2:**
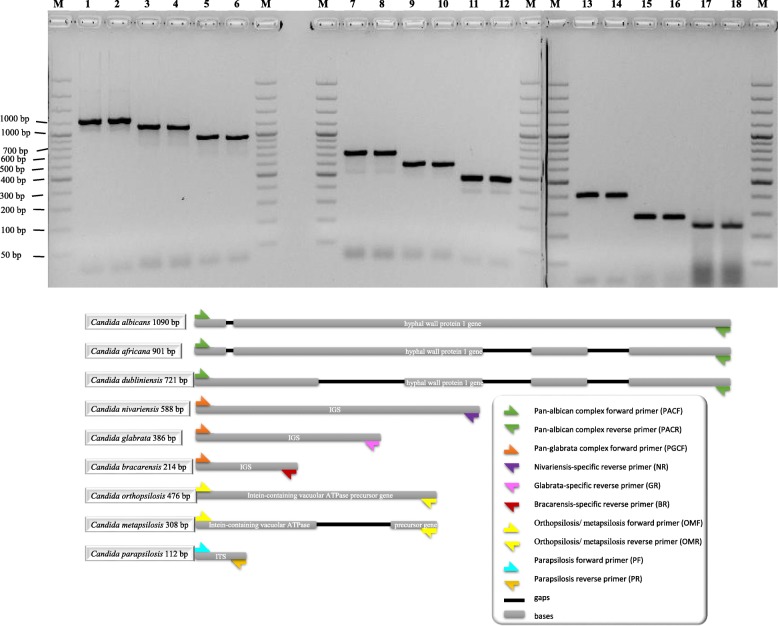
Amplified PCR products for nine target cryptic species complexes are shown. M. 100 bps Molecular ladder (SM 0324), 1–2 *C. albicans* (1090 bps), 3–4 *C. africana* (901 bps), 5–6 *C. dubliniensis* (721 bps), 7–8 *C. nivariensis* (586 bps), 9–10 *C. orthopsilosis* (476 bps), 11–12 *C. glabrata* (386 bps), 13–14 *C. metapsilosis* (308 bps), 15–16 *C. bracarensis* (214 bps), 17–18 *C. parapsilosis* (112 bps)

Subjecting 111 CBS reference strains belonging to the species complexes of *C. albicans* (*n* = 55), *C. glabrata* (*n* = 28), and *C. parapsilosis* (*n* = 28) successfully differentiated and identified the nine target species (Fig. 2). Although, the lengths of PCR products were different from those were designed, target species were unequivocally distinguishable from one another (Figs. 1 and 2 and Table 3). In order to prevent misidentifications it is advised to prepare in-house master mixes in large scale by mixing PCR products of target species and storing them at 4 °C for future identification purposes.

**Table 3:** Expected and actual PCR products on gel are shown. Despite of deviation of amplicon sizes in both individual PCR products and home-made ladder containing mixture of PCR products, target species were clearly distinguishable from each other

Target species	Expecetd PCR product size	Actual home-made ladder PCR product sizes on gel (approximately)	Actual indidividual PCR product size on gel (approximately)
*Candida albicans*	1090 bps	950 bps	1200 bps
*Candida africana*	901 bps	800 bps	1100 bps
*Candida dubliniensis*	721 bps	600 bps	950 bps
*Candida nivariensis*	588 bps	500 bps	700 bps
*Candida orthopsilosis*	476 bps	&gt; 400 bps	576 bps
*Candida glabrata*	386 bps	&gt; 350 bps	486 bps
*Candida metapsilosis*	308 bps	˜280 bps	400 bps
*Candida bracarensis*	214 bps	˜200 bps	300 bps
*Candida parapsilosis*	112 bps	100 bps	250 bps

### Specificity testing using the blind test set

Subjecting 100 DNA samples (containing 66 fungal species) to our 9-plex PCR assay ranging from closely- and distantly-related yeast species, *Aspergillus* spp. resulted in 100% specificity and no cross-reaction with any other fungal species. Additionally, subjecting human DNA to our 9-plex PCR assay, showed no cross-reaction either.

### Pure colony testing

Subjecting 105 yeast pure colonies from 61 yeast species pooled from the optimization test set (Additional file 1: Table S1) resulted in successful amplification of all nine target species. No PCR inhibition by cell components was observed, indicating that pure colonies are compatible with the 9-plex PCR. Compatibility of our 9-plex PCR with pure colony testing allowed decreasing turn-around time from 7 h to 4 h.

### Agreement of 9-plex PCR assay with MALDI-TOF MS

Results obtained from our 9-plex PCR for 280 clinical isolates were compared with MALDI-TOF (Table 4). Previously in Westerdijk Fungal Biodiversity Institute, clinically isolated yeast species were identified by MALDI-TOF MS and it showed 98–100% agreement with sequencing [19, 20]. Hence, in this study MALDI-TOF MS along with Sanger sequencing were used as validation tools. Additionally, the spectra for many yeast species utilized in brewery have been added to the MALDI-TOF CBS in-house database. Of 131 strains identified as *C. albicans* using MALDI-TOF MS, our 9-plex PCR assay identified 128 as *C. albicans* and three *as C. africana*. The rest of cryptic species complexes of *C. glabrata* (*n* = 91), and *C. parapsilosis* (*n* = 54) were all identified correctly by MALDI-TOF MS. The overall agreement between MALDI-TOF MS and our 9-plex PCR assay was 98.93%.

**Table 4:** Comparison of 9-plex PCR with CBS in-house MALDI-TOF database and sequencing of D1/D2 LSU rDNA

Species	Multiplex PCR	MALDI-TOF	Sequencing
*C. albicans* (*n* = 128)	*C. albicans* (*n* = 128)	*C. albicans* (*n* = 128)	*C. albicans* (*n* = 128)
*C. dubliniensis* (*n =* 4)	*C. dubliniensis* (*n =* 4)	*C. dubliniensis* (*n =* 4)	*C. dubliniensis* (*n =* 4)
*C. africana* (*n = 3*)	*C. africana* (*n = 3*)	*C. albicans* (*n = 3*)	*C. africana* (*n = 3*)^a^
*C. glabrata* (*n =* 87)	*C. glabrata* (*n =* 87)	*C. glabrata* (*n =* 87)	*C. glabrata* (*n =* 87)
*C. nivariensis* (*n =* 2)	*C. nivariensis* (*n =* 2)	*C. nivariensis* (*n =* 2)	*C. nivariensis* (*n =* 2)
*C. bracarensis* (*n =* 2)	*C. bracarensis* (*n =* 2)	*C. bracarensis* (*n =* 2)	*C. bracarensis* (*n =* 2)
*C. parapsilosis* (*n =* 28)	*C. parapsilosis* (*n =* 28)	*C. parapsilosis* (*n =* 28)	*C. parapsilosis* (*n =* 28)
*C. orthopsilosis* (*n =* 6)	*C. orthopsilosis* (*n =* 6)	*C. orthopsilosis* (*n =* 6)	*C. orthopsilosis* (*n =* 6)
*C. metapsilosis* (*n =* 20)	*C. metapsilosis* (*n =* 20)	*C. metapsilosis* (*n =* 20)	*C. metapsilosis* (*n =* 20)

^a^ Similarity between *C. albicans* and *C. africana* using sequencing of D1/D2 LSU rDNAwas more than 99%

This library has been enriched with a diverse range of yeast species utilized in brewery

### Agreement of 9-plex PCR assay with sequencing

Results obtained from our 9-plex PCR was 100% consistent with sequencing of D1/D2 domains of LSU rDNA for 280 clinical strains (Table 4). Cryptic species complexes of *C. parapsilosis* (*n* = 54) and *C. glabrata* (*n* = 91) were all clearly distinguishable using sequencing of respective domain. Despite the fact that, *C. dubliniensis* (*n* = 4) and *C. albicans* (*n* = 128) clearly were distinguishable using sequencing of respective domain, *C. africana* and *C. albicans* showed &gt; 99% similarity, and, hence, making their discrimination at the species level difficult. However, our 9-plex PCR assay clearly discriminated the two closely related species complexes of *C. albicans* and *C. africana*. Consistently, 9-plex PCR assay distinctly identified all of the cryptic species complexes as distinct fragment on the gel. The overall agreement between sequencing of D1/D2 LSU rDNA was 100%.

## Discussion

Representatives of the cryptic species of *Candida albicans*, *Candida glabrata*, and *Candida parapsilosis* complexes account for the majority of candidiasis cases [21]. Appropriate identification and differentiation of cryptic species complexes is clinically relevant, as not only there are differences on virulence and antifungal susceptibility patterns among species within the same complex, but also contradictory observations for antifungal susceptibility patterns among different studies have been reported [5, 22–25]. Due to facing these contradictory results along with the limited epidemiological data for cryptic species complexes, the genuine distribution and antifungal susceptibility profiles of these species in different geographical locations remained unclear. Moreover, as antifungal susceptibility profile of cryptic species within the same complex are varied, identification down to the species level is imperative to establish the appropriate antifungal therapy [24, 26, 27]. In line with this, providing epidemiologists and small laboratories with a fast, accurate, specific, and cheap means of identification to disclose the prevalence and antifungal susceptibility profiles of isolates belonging to cryptic *Candida* species. Accordingly, we have developed and validated an inexpensive, reliable, accurate, specific, and user friendly multiplex PCR assays capable of identifying nine cryptic species in one assay.

From different perspectives including, time needed to finish the experiments, required expenses and need of trained technicians our assays is comparable to other PCR-based assay and platforms such as MALDI-TOF.

Application of cryptic species complexes strains isolated in clinical settings with our 9-plex PCR assay and its comparison with results obtained by Sanger sequencing of D1/D2 domain of LSU rDNA, revealed 100% consistency between these techniques. However, due to the high similarity between *C. albicans* and *C. africana* in the sequences of the D1/D2 and ITS rDNA fragments (99.3–100%), distinguishing these two species is difficult [28]. Consistently, the sequencing of LSU rDNA fragment in *C. africana* showed &gt; 99% similarity with *C. albicans*. Importantly, application of our 9-plex PCR could discriminate these two species. Like DNA microarray [29] and pyrosequencing [30], Sanger sequencing requires highly trained technicians and more turn-around time [8], while our multiplex PCR assay is straightforward and running of the whole application (from master mix preparation to visualization on the gel) only consumes four hours. In terms of expenses, only 0.75–1 euro is enough to finalize the results on the gel electrophoresis, however, sequencing require specific devices and is more expensive. However, in order to resolve the issue of discrepancy between the length of predicted and actual PCR products we suggest to use either our in-house ladder or to run the amplicons of each control species individually in a separate lane.

Our multiplex PCR successfully identified all *C. africana* isolates and, hence, improved the results obtained from MALDI-TOF. Except for *C. africana* strains (*n =* 3), Bruker MALDI-TOF MS could identify the rest of cryptic species complexes, resulting in 98.93% agreement with our multiplex PCR assay. Variability in accuracy of commercial MALDI-TOF MS database for identification of uncommon and cryptic *Candida* species [9, 31, 32], inability of the Bruker MALDI-TOF to distinguish *C. africana* from *C. albicans*, and incompetence of VITEK MS systems for identification of *C. bracarensis*, *C. nivariensis*, and *C. orthopsilosis* and low cut-off value of Bruker MS systems (&lt; 1.700) for identification of *C. bracarensis* complex reinforced the urge for molecular identification tools [22, 33]. On the other hand, unlike the domination of PCR even in developing countries [12, 13], MALDI-TOF is a newer introduced platform, mainly restricted to large or reference laboratories [34–36].

Unlike, AFLP [9] and RFLP [37], there is no need for restriction of the PCR products, several visualization steps and reading of the results are straightforward. Muriel Cornet et al. (2011) used PCR-RFLP, to identify all eight cryptic species except for *C. africana*. However, this method used three primers targeting ribosomal intergenic spacer (IGS), tedious post-PCR restriction and required electrophoretic visualization twice making this experiment daunting, time consuming, and expensive [37]. Additionally, digestion of PCR products with restriction enzymes generated multiple fragments, in turn, makes the interpretation difficult when compared to banding pattern of reference strains.

The successful identification of all nine cryptic species in one tube, complements previous studies identifying members of the species complexes of *Candida albicans* [14], *Candida glabrata* [15], and *Candida parapsilosis* [16] using three separate tubes, and, hence, is time- and cost-saving.

Application of broad diversity of yeast species, five *Aspergillus* species, and human DNA for specificity testing and inclusion of an extensive number of CBS reference strains and clinical isolates, showed that our 9-plex PCR is 100% specific. As a result, our assay circumvents the imperfections of phenotypic assays (CHROMagar, VITEK 2 and ID 32C) that suffer from the lack of specificity [38, 39]. Additionally, subjecting various populations of the same species to phenotypic assay could showed various results [32, 40, 41]. Accordingly, as suggested by Griseo et al. (2015), small clinical laboratories can take advantage of specific phenotypic methods supplemented with easy-to-perform PCR-based approaches to identify and report isolated cryptic *Candia* species [8].

## Conclusion

Due to the problems with the identification and discrimination of cryptic *Candida* species from their closest relatives, there is still uncertainty and unclarity about their epidemiology, pathogenicity and antifungal susceptibility pattern [43,44]. Consequently, developing reliable, specific, cost and labor effective methods is necessary. Successful testing of specificity and validation using a broad range of CBS reference strains and clinical isolates, revealed the potential of this assay to be implemented in routine diagnostics and epidemiological studies. As *C. albicans*, *C. glabrata*, and *C. parapsilosis* complex species constitute 80–90% of candidiasis cases, identification of all nine cryptic species within one multiplex PCR assay, could be of a great assistance.

## Additional files

Additional file 1: **Table S1.** CBS reference strains utilized for optimization of 9-plex PCR. (DOCX 20 kb)

Additional file 2: **Figure S1.** Comparison of home-made ladder and Thermofisher commercial ladder (SM0323). Obviously, amplicons of all target species are distinguished and differentiated from one another. (JPG 79 kb)
