# Supplementary material for: Identification of nine cryptic species of Candida albicans, C. glabrata, and C. parapsilosis complexes using one-step multiplex PCR
Source: BMC Infect Dis. 2018 Sep 25;18:480. doi: 10.1186/s12879-018-3381-5 (PMC6156947; doi:10.1186/s12879-018-3381-5)
Supplement: Supplementary file 1 — Table S1. CBS reference strains utilized for optimization of 9-plex PCR. (DOCX 20 kb) [file 12879_2018_3381_MOESM1_ESM.docx]

Table S1. CBS reference strains utilized for optimization of 9-plex PCR

| **Species** | **Strain number (n=168)** |
| --- | --- |
| **Cryptic *Candida* species** | **111** |
| *C. albicans* | 40 |
| *C. africana* | 5 |
| *C. dubliniensis* | 10 |
| *C. glabrata* | 20 |
| *C. bracarensis* | 5 |
| *C. nivariensis* | 3 |
| *C. parapsilosis* | 20 |
| *C. metapsilosis* | 5 |
| *C. orthopsilosis* | 3 |
| **Closely- and distantly-related species** | **57** |
| *Pichia kudriavzevii* | CBS 5147 |
| *Kluyveromyces marxianus* | CBS 607 |
| *Pichia guilliermondii* | CBS 7099 |
| *Clavispora lusitaniae* | CBS 7270 |
| *Debaromyces hansenii* | CBS 796 |
| *C. norvegensis* | CBS 1922 |
| *Yarrowia lipolytica* | CBS 6124 |
| *C. castellii* | CBS 4332 |
| *C. viswanathii* | CBS 4024 |
| *C. utilis* | CBS 841 |
| *Kluyveromyces lactis* | CBS 845 |
| *C. inconspicua* | CBS 2833 |
| *C. auris* | CBS 10913 |
| *C. haemulonii* | CBS 5149 |
| *C. duobushaemulonii* | CBS 7798 |
| *C. pseudohaemulonii* | CBS 10004 |
| *C. sake* | CBS 159 |
| *C. humilis* | CBS 5658 |
| *C. membranifaciens* | CBS 1952 |
| *C. pararugosa* | CBS 1010 |
| *C. rugosa* | CBS 613 |
| *C. maltosa* | CBS 5611 |
| *C. intermedia* | CBS 572 |
| *C. magnoliae* | CBS 166 |
| *C. infanticola* | CBS 7922 |
| *Schizosaccharomyces pombe* | CBS 356 |
| *C. stellatoidea* | CBS 1905 |
| *C. zeylanoides* | CBS 619 |
| *Candida norvegica* | CBS 4239 |
| *Metschnikowia pulcherrima* | CBS 5833 |
| *Meyerozyma caribbica* | CBS 9966 |
| *Saccharomyces cerevisiae* | CBS 1171 |
| *Kodamaea ohmeri* | CBS 5367 |
| *Lodderomyces elongisporus* | CBS 2605 |
| *Pichia cactophila* | CBS 6926 |
| *Pichia fermentans* | CBS 187 |
| *Pichia kluyveri* | CBS 188 |
| *Exophiala dermatitidis* | CBS 207.35 |
| *Hortaea werneckii* | CBS 107.67 |
| *Galactomyces geotrichum* | CBS 772.71 |
| *Wickerhamomyces anomalus* | CBS 5759 |
| *Zygosaccharomyces rouxii* | CBS 732 |
| *Lindnera fabianii* | CBS 5640 |
| *Magnusiomyces capitatus* | CBS 162.80 |
| *Malassezia pachydermatis* | CBS 1879 |
| *Rhodotorula mucilaginosa* | CBS 316 |
| *Torulaspora globosa* | CBS 764 |
| *Trichosporon asahii* | CBS 2479 |
| *Trichosporon inkin* | CBS 5585 |
| *Cryptococcus gattii* | CBS 6955 |
| *Cryptococcus neoformans* | CBS 6885 |
| *Cryptococcus deneoformans* | CBS 132 |
| *Aspergillus fumigatus* | CBS 133.61 |
| *Aspergillu niger* | CBS 554.65 |
| *Aspergillu terreus* | CBS 601.65 |
| *Aspergillu nidulans* | CBS 589.65 |
