# Supplementary figures and images for: Identification of nine cryptic species of Candida albicans, C. glabrata, and C. parapsilosis complexes using one-step multiplex PCR
Source: BMC Infect Dis. 2018 Sep 25;18:480. doi: 10.1186/s12879-018-3381-5 (PMC6156947; doi:10.1186/s12879-018-3381-5)

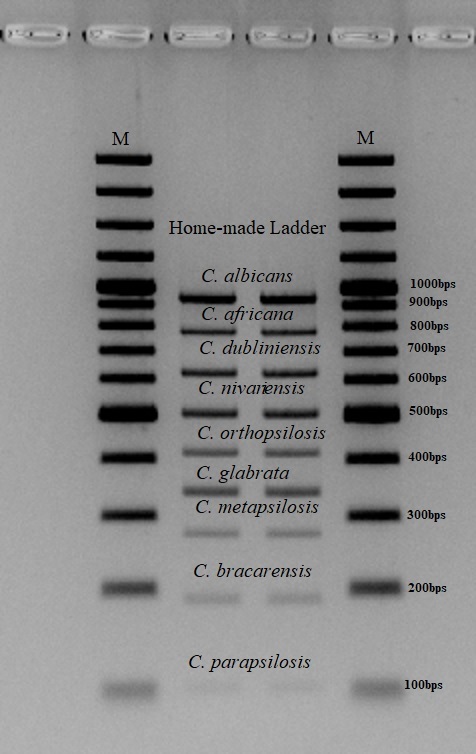

Supplement: Supplementary file 2 — Figure S1. Comparison of home-made ladder and Thermofisher commercial ladder (SM0323). Obviously, amplicons of all target species are distinguished and differentiated from one another. (JPG 79 kb) [file 12879_2018_3381_MOESM2_ESM.jpg]
